# Supplementary material for: Yeast Cip1 is activated by environmental stress to inhibit Cdk1–G1 cyclins via Mcm1 and Msn2/4
Source: Nat Commun. 2017 Jul 4;8:56. doi: 10.1038/s41467-017-00080-y (PMC5496861; doi:10.1038/s41467-017-00080-y)
Supplement: Supplementary file 1 — Supplementary Information [file 41467_2017_80_MOESM1_ESM.pdf]

Type of file: PDF
Title of file for HTML: Supplementary Information
Description: Supplementary Figures, Supplementary Tables and Supplementary References

Type of file: PDF
Title of file for HTML: Peer Review File
Description:

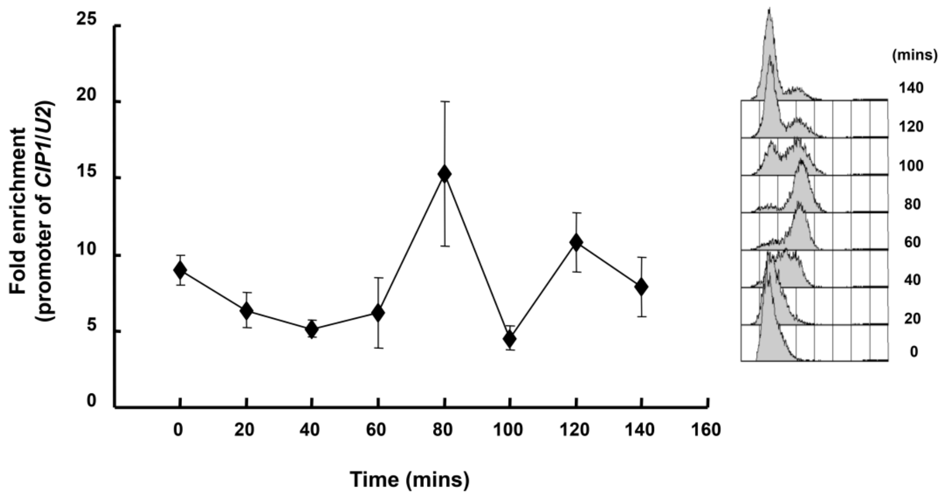

Supplementary Figure 1. Mcm1 binds to the *CIP1* promoter at late M phase.

Wild-type cell was first synchronized at G1 phase and released by subsequently removing α-factor from the cultures. After releasing from G1, cells were collected at indicated time points, and α-factor was re-added into the medium at 60 mins. The binding of Mcm1 on the *CIP1* promoter was determined by ChIP and the fold enrichment relative to time point 0 of Mcm1 binding was calculated. The DNA content of cells at each time point was analyzed by FACS.

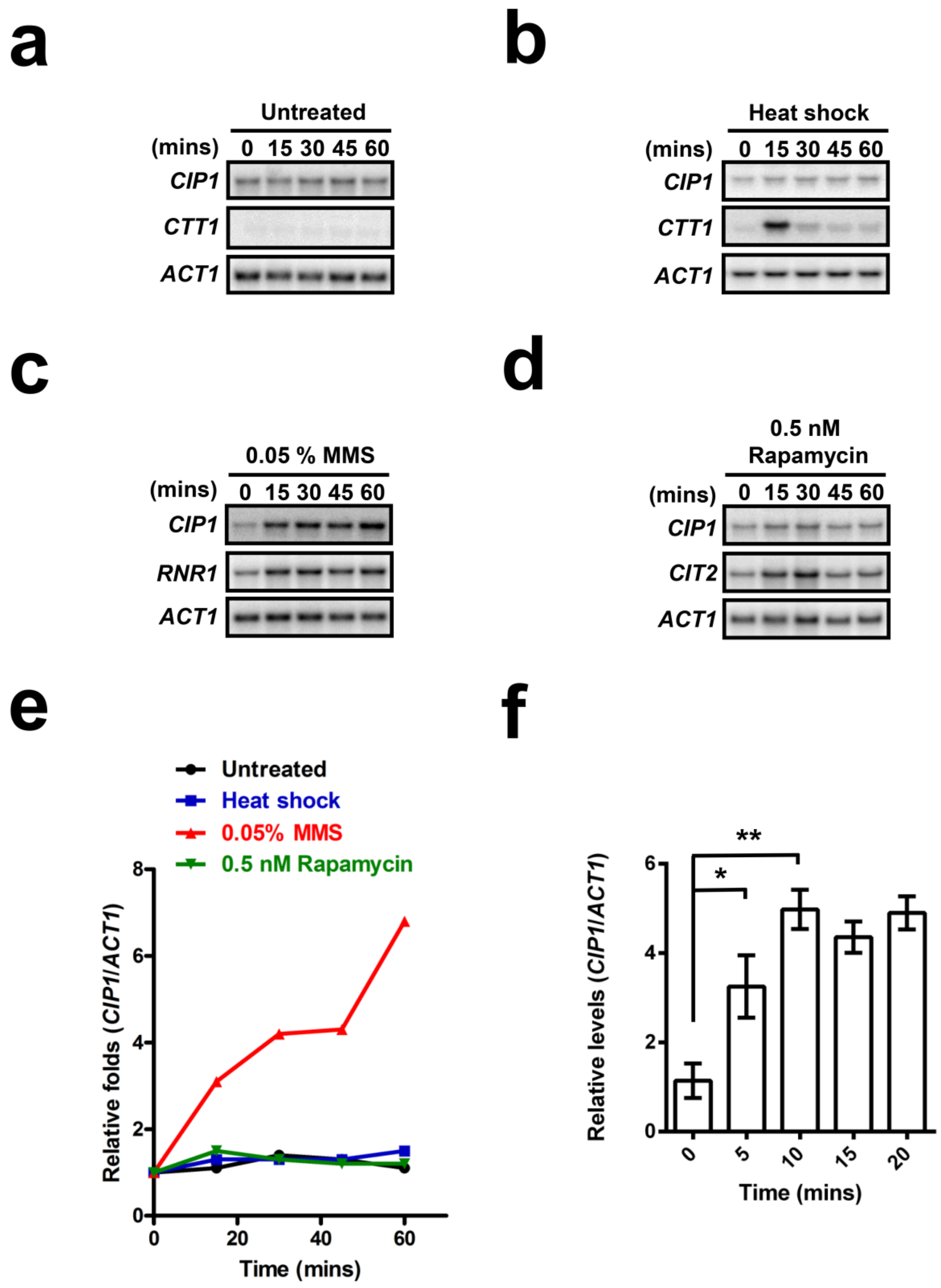

Supplementary Figure 2. ***CIP1*** **expression is induced under several stresses.**

(a-d) Yeast cells were treated with various stresses including (a) untreated, (b) heat (37ºC), (c) MMS (0.05 %), and (d) rapamycin (0.5 nM). Samples were collected at the indicated time points after stress treatment. Total RNA was analyzed by Northern

blotting and hybridized with indicated probes. *CTT1* referred to the positive control of these stresses, and *ACT1* was used as a loading control. (e) The band intensities displayed in the broken-line graph of each panel were quantified using Image J, normalized relative to respective internal controls, and expressed as the ratio of the *CIP1* levels to the time point 0 at the beginning of each stress treatments. The values were given as mean ± S.D. (n=3). mRNA expression was considered to be upregulated when relative fold exceeds 2. (f) Yeast cells were treated with osmotic stress. Samples were collected at indicated time points after 0.5 M KCl treatment. *CIP1* expression was analyzed by qRT-PCR and normalized to *ACT1*. The values were given as mean ± S.D. (n=3, *P<0.05, **P< 0.01, Student's t-test, two tailed).

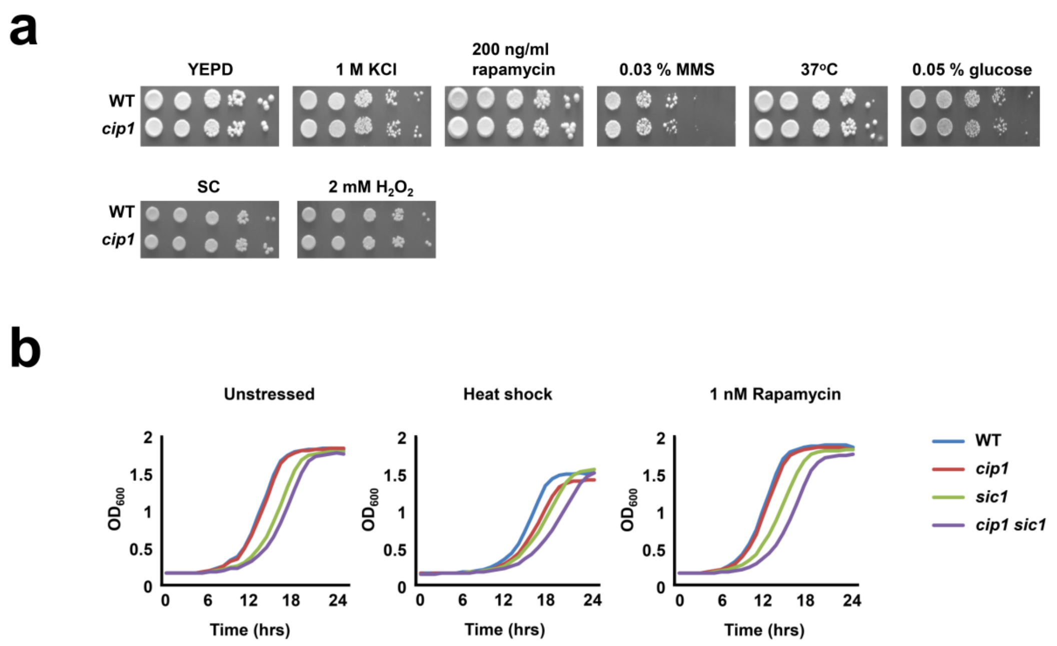

Supplementary Figure 3. *CIP1* single deleted cells are not sensitive to stresses.

(a) Exponentially growing cells were spotted in 10-fold diluted equal number of yeast on YEPD or SC plates containing the indicated stresses and incubated at 30°C. For heat shock, the plate was incubated at 37°C. (b) Exponentially growing cells were diluted to an OD_{600}=0.005 and grown in YEPD. Cells were treated with unstressed control (30°C), heat stress (37°C), and 1nM Rapamycin (30°C). OD measurements were estimated every hour for 25 hours in a 96-well plate using Synergy H1 Multi-Mode Reader.

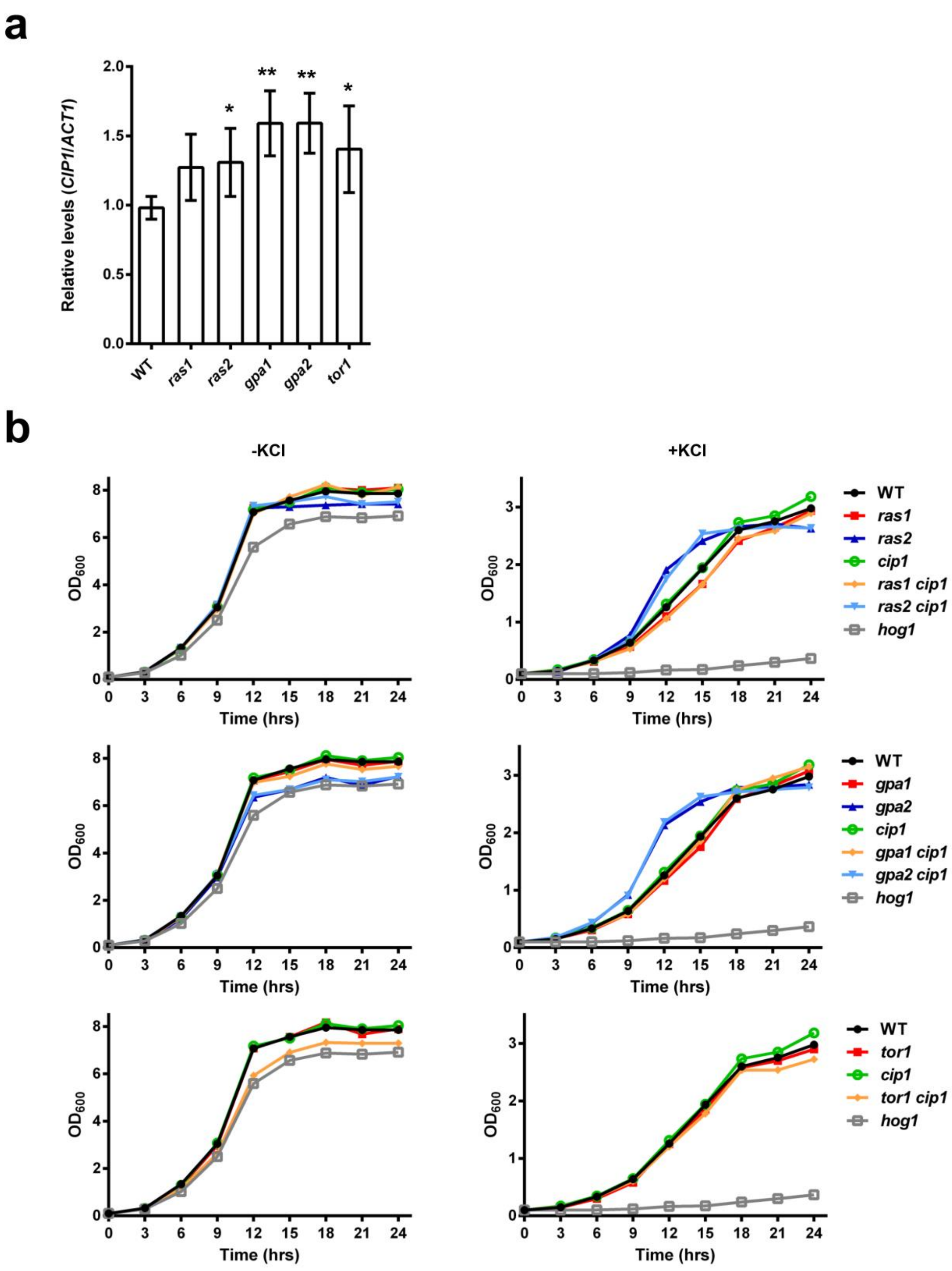

Supplementary Figure 4. Deletion of Msn2/4 upstream suppressors slightly increases *CIP1* expression in the absence of stress.

(a) Exponentially growing cells containing deletion of different Msn2/4 upstream suppressors (*ras1*, *ras2*, *gpa1*, *gpa2*, *tor1*, *bck1* and *slt2*) were collected. *CIP1*

expression was analyzed by qRT-PCR and normalized to *ACT1*. The values were given as mean ± S.D. (n=4). The significant difference of each deleted strain was compared to WT cells. (*P<0.05, **P< 0.01, Student's t-test, two tailed). (b) Growth curves of cells containing deletion of different Msn2/4 upstream suppressors. Cells were diluted to an OD_{600}=0.1 and grown in SC medium with or without 1.2 M KCl. OD measurements were estimated every three hours for 24 hours.

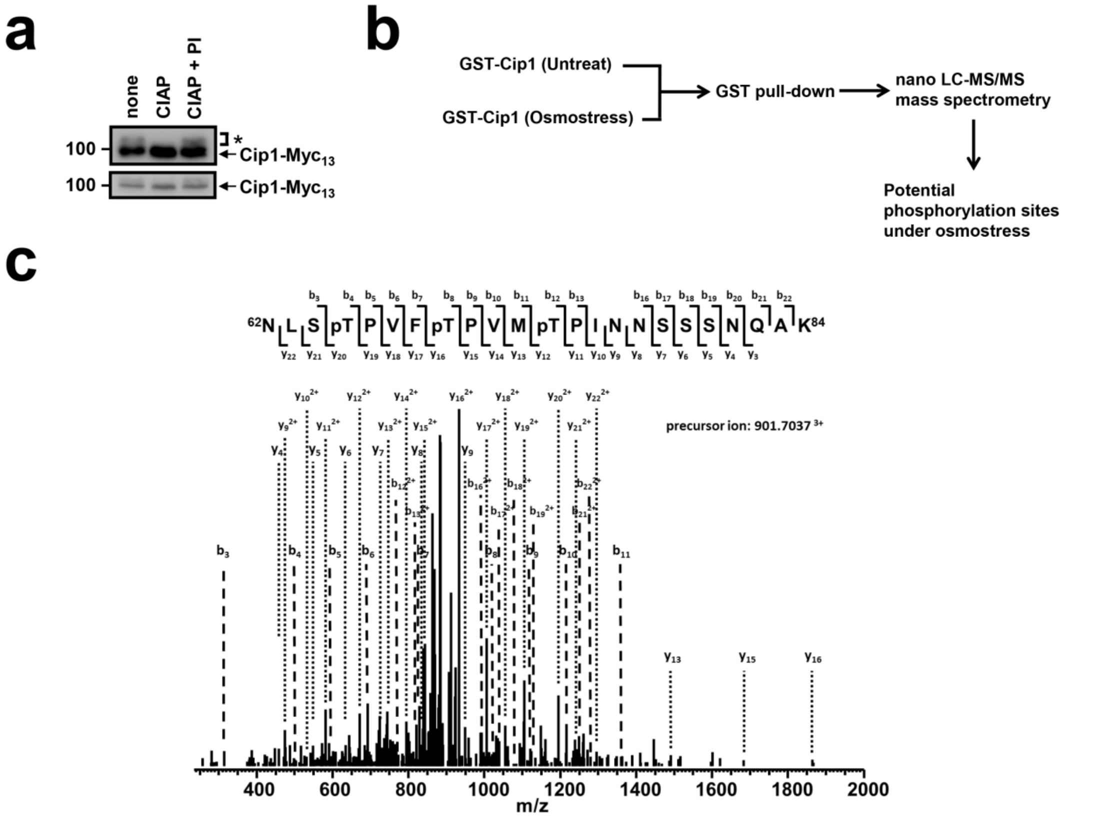

Supplementary Figure 5. Mass analysis of the phosphorylation on Cip1.

(a) Western analysis of C-terminal tagged Cip1-Myc$_{13}$ from KCl treated cells using anti-Myc antibody. The treatment of CIAP phosphatase removed the phosphorylation signal. The phosphorylated Cip1 was indicated by asterisk (*) in the upper panel (7% SDS-PAGE) and the lower panel (12% SDS-PAGE) serves as a loading control. PI, phosphatase inhibitor. (b) Schematic diagram of the LC-MS/MS experimental process and computational approach for phosphorylation identification and quantitation on Cip1 under osmotic stress. (c) GST-tagged Cip1 was overexpressed under hyperosmotic stress, immunoprecipitated from cells, trypsin-digested and subjected to LC-MS/MS analysis.

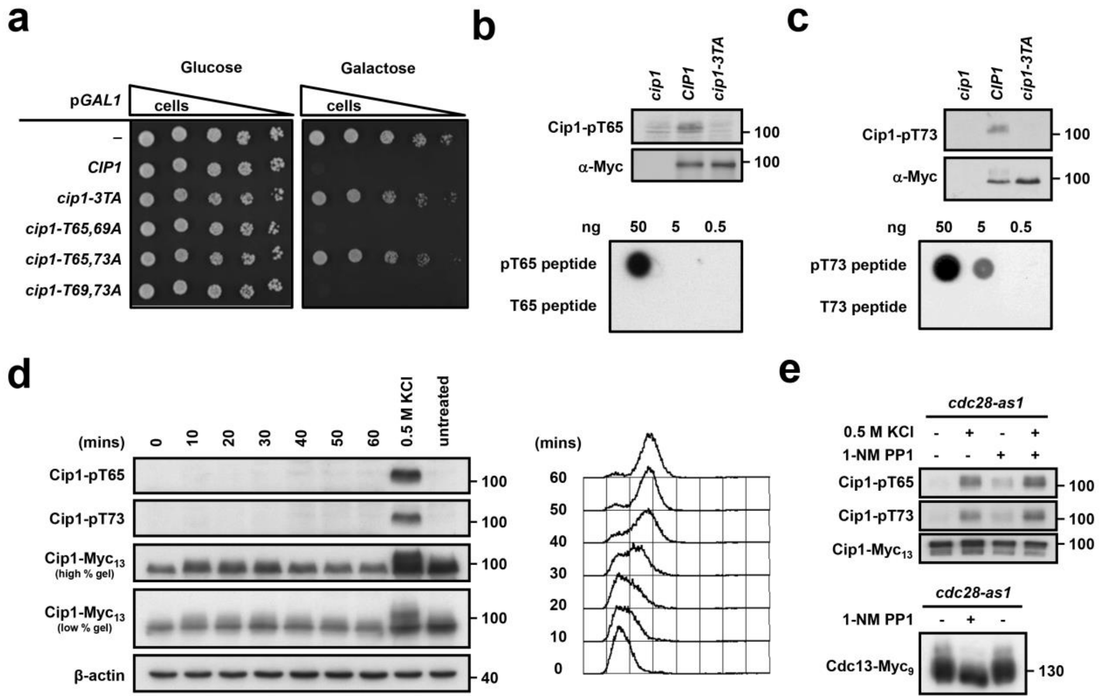

Supplementary Figure 6. Characterization of the phosphor-specific antibodies against Cip1 T65 and T73.

(a) Wild-type cells bearing empty vector, *GAL1-CIP1*, *GAL1-cip1-3TA*, *GAL1-cip1-T65, 69A*, *GAL1-cip1-T65, 73A*, or *GAL1-cip1-T69, 73A* plasmid were spotted in 10-fold diluted equal number of yeast on 2 % glucose and 2% galactose plates. (b, c) Upper panel: Western analysis of endogenous C-terminal tagged Cip1-Myc$_{13}$ from 0.5M KCl treated cells using Cip1 pT65 and pT73 specific antibodies. The Myc antibody detected Cip1-Myc$_{13}$ was used as a loading control. Lower panel: The phosphor-specific antibody against Cip1 pT65 and pT73 recognized the phosphor-peptides of Cip1 in dot blot assay. (d) Cell cycle independent phosphorylation of Cip1. The overnight culture was grown to early log phase in YEPD, arrested at G1 by α-factor, and released into cell cycle. Cells were collected at

10-minute intervals at 30°C for 90 mins, and Cip1 was analyzed by Western blot analysis using Cip1 pT65 or pT73 specific antibodies. The mobility shift of Cip1 phosphorylation was shown in low percentage gel. The anti-Myc antibody was used against endogenous total Cip1 and β-actin was served as internal control. The FACS analysis of different time points was shown at the right. (e) W303 (WT) and *cdc28-as1* were grown to exponential phase and treated with or without 0.5μM 1-NM PP1 for 3 hours. Cells were then subjected to osmostress (0.5 M KCl) for 15 mins. Lysates were extracted, and Cip1 phosphorylation was analyzed by Western blot analysis. As previously described[1] a Cdk1 substrate, Cdc13, was served as a positive control.

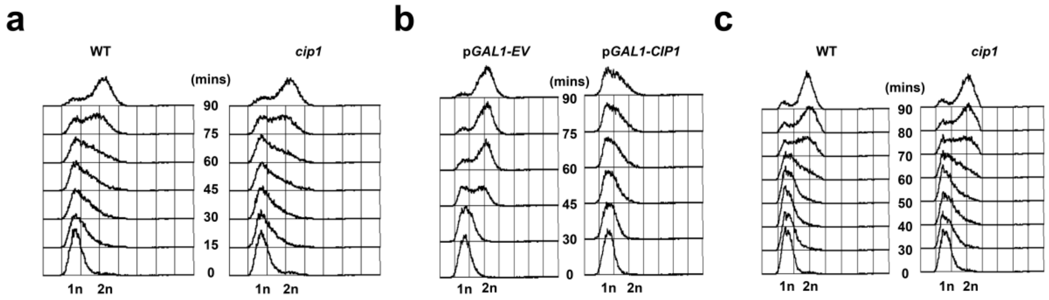

Supplementary Figure 7. Cell cycle progression in Cip1 overexpressed or deleted cells.

(a) The DNA content of strains in Fig. 6d was analyzed by FACS analysis. α-factor synchronized wild-type and *cip1Δ* cells were released in medium containing 0.5M KCl at 24ºC. At 15-minute intervals, cells were collected for FACS analysis and some were cultivated in trapping medium for further analysis. (b) In Fig. 7d, strains with empty vector or *GAL1-CIP1* were first arrested at G1 phase by α-factor in 2% raffinose. Cip1 was induced in the present of 2% galactose. The α-factor was subsequently removed from the cultures and samples were collected at the indicated time points at 30ºC for FACS analysis. (c) Strains in Fig. 7e were α-factor arrested and released into medium containing 0.5M KCl at 24ºC. Cells were collected at 10-minute intervals for FACS analysis.

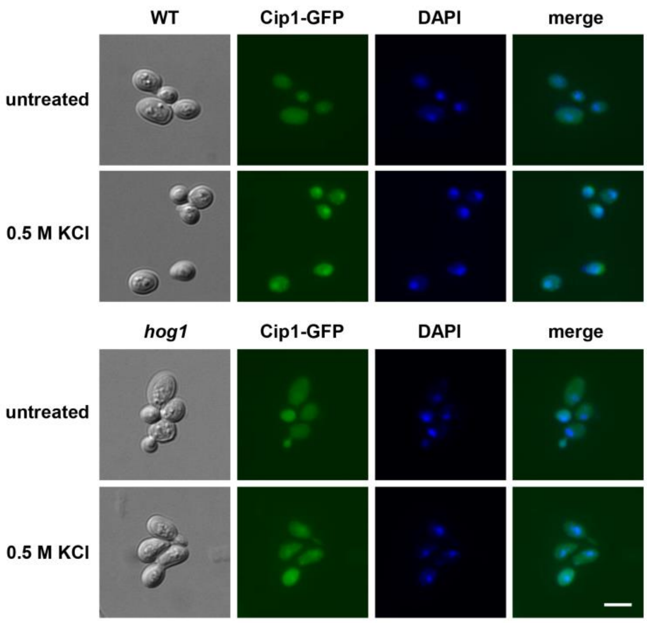

Supplementary Figure 8. Cip1 localizes to the nucleus after osmotic stress.

WT and *hog1* strains overexpressing *CIP1-GFP* from a *leu2d* plasmid were grown to exponential phase in SC-uracil medium. Cip1-GFP was induced in SC-leucine medium at 30°C for 3 hours. Cells were collected before (untreated) and after the addition of 0.5 M KCl. Cip1-GFP was detected by fluorescence microscopy and nuclei were visualized using DAPI (Scale bar, 5 μm).

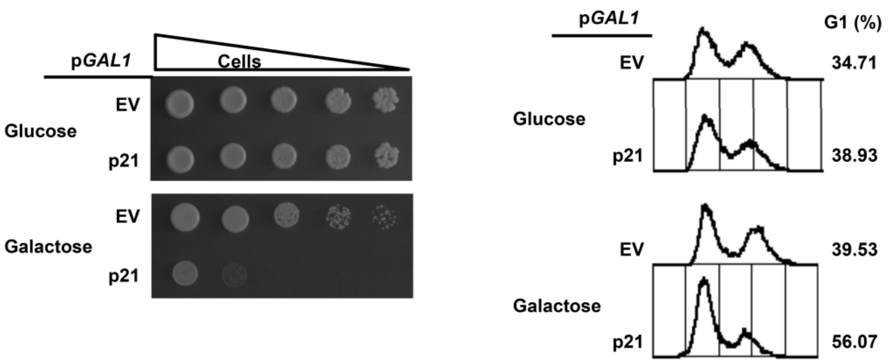

Supplementary Figure 9. Human p21 expression stimulates G1 arrest in yeast cells.

Isogenic WT strains bearing an empty vector or a *GAL1*-p21 plasmid were spotted in 10-fold diluted equal number of yeast on 2 % glucose and 2% galactose plates. The DNA content of these strains was shown at the right by FACS analysis, and the percentage of G1 cells was calculated.

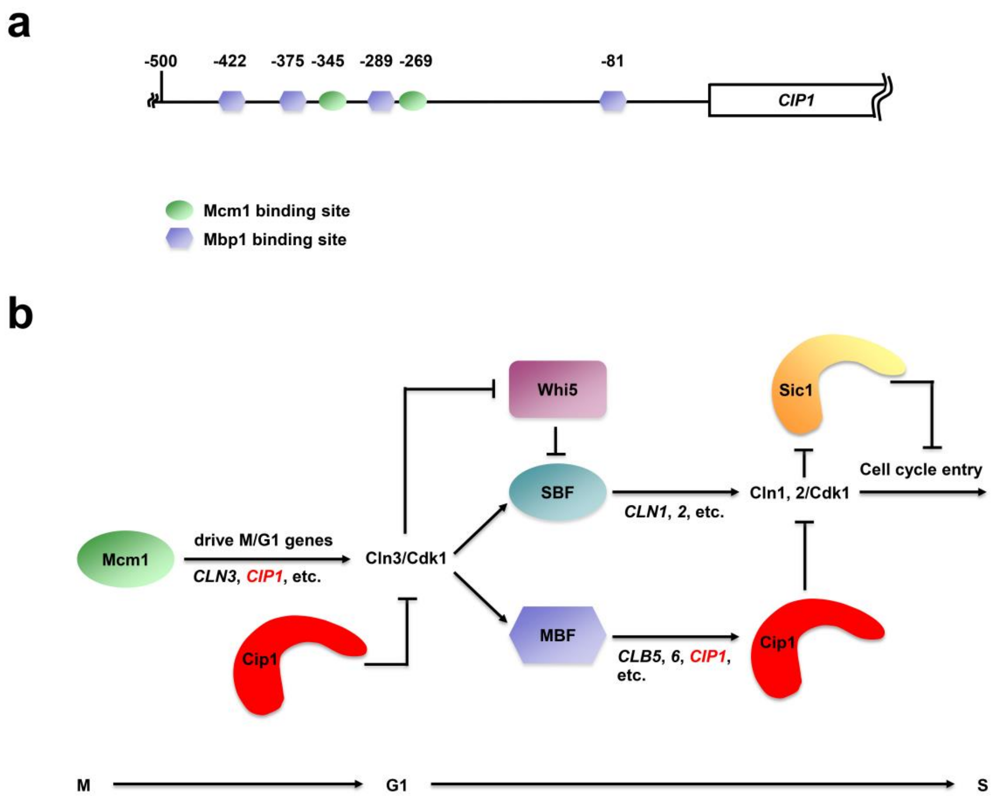

Supplementary Figure 10. Mcm1 and Mbf1 drive *CIP1* transcription at different cell cycle stages.

(a) The DNA binding motifs for Mcm1 (shown in gray) and Mbp1 (shown in black) at the *CIP1* promoter. (b) Model of *CIP1* transcriptional regulation. Activation of Mcm1-dependent transcription results in the initial accumulation of *CIP1* mRNA and the expressed Cip1 mainly inhibits Cdk1-Cln3 complex activity at early G1 phase. In late G1 phase, Cdk1-Cln3 complex promotes the inhibitory phosphorylation of Whi5 to unleash active SBF and MBF (Swi6-Mbp1). Active MBF transcribes *CIP1* mRNA and the expressed Cip1 at late G1 mainly targets to Cdk1-Cln1, 2 complexes to block S phase entry. On the other hands, active Cdk1-Cln1, 2 complexes inhibit the S phase

inhibitor, Sic1, to promote cell cycle entry.

Figure 1e

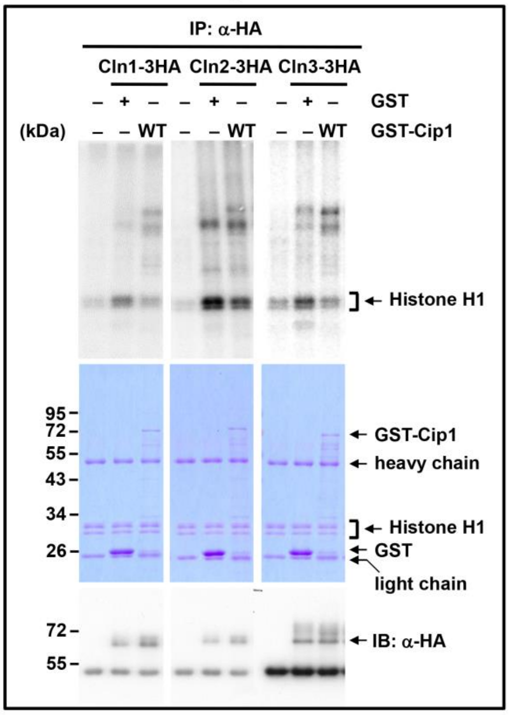

Figure 2a

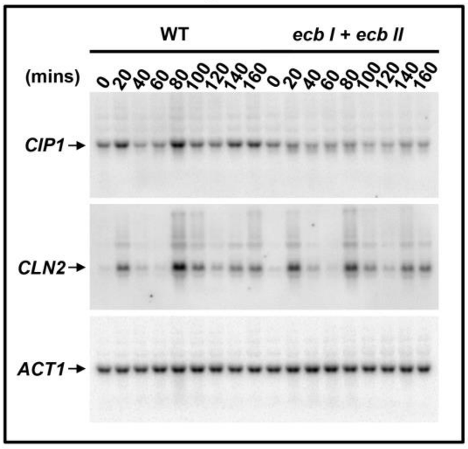

Figure 3a

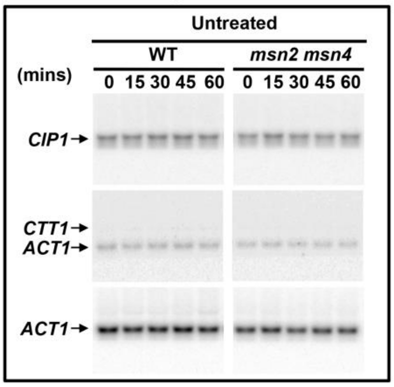

Figure 3b

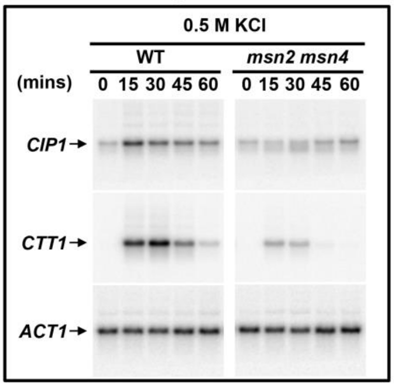

Figure 3c

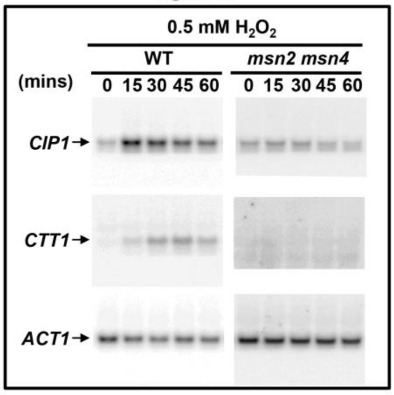

Figure 3d

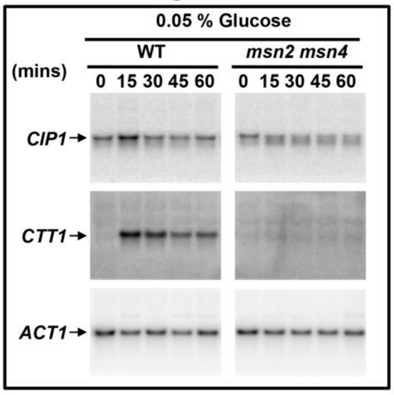

Figure 4d

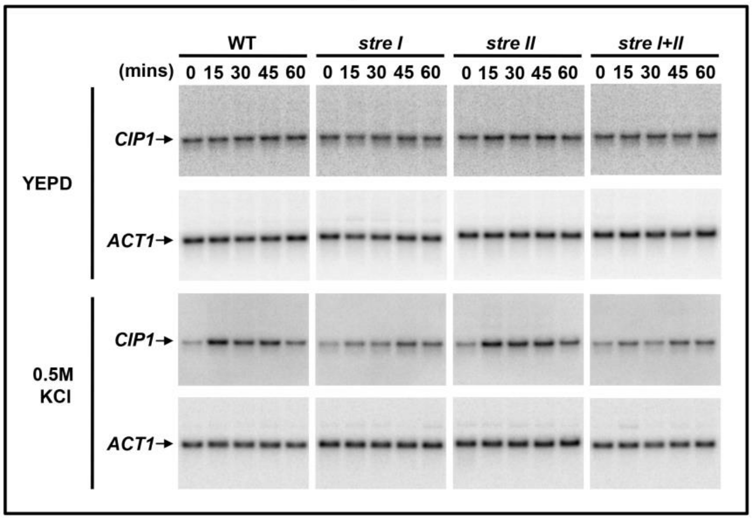

Figure 5c

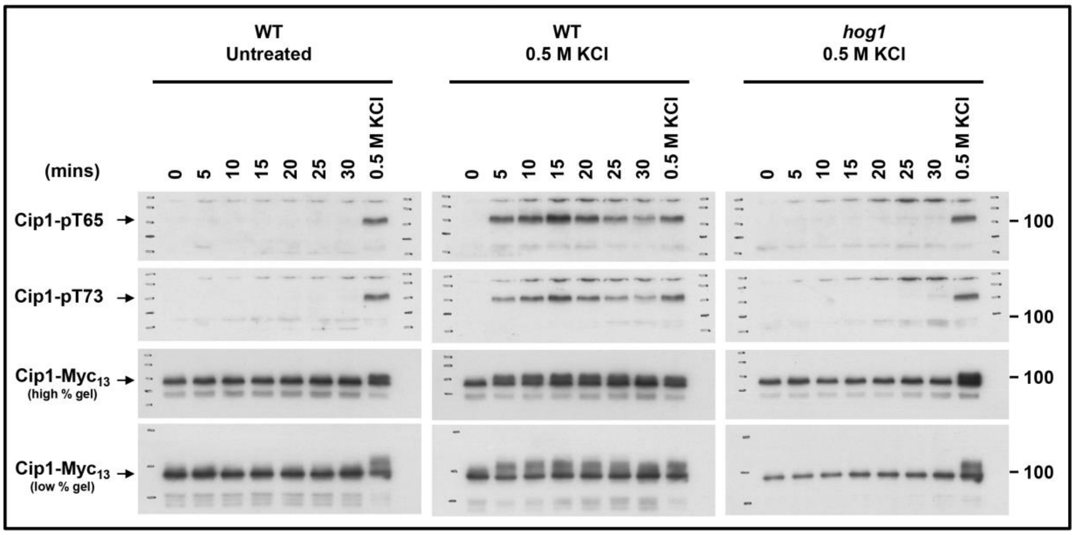

Figure 7a

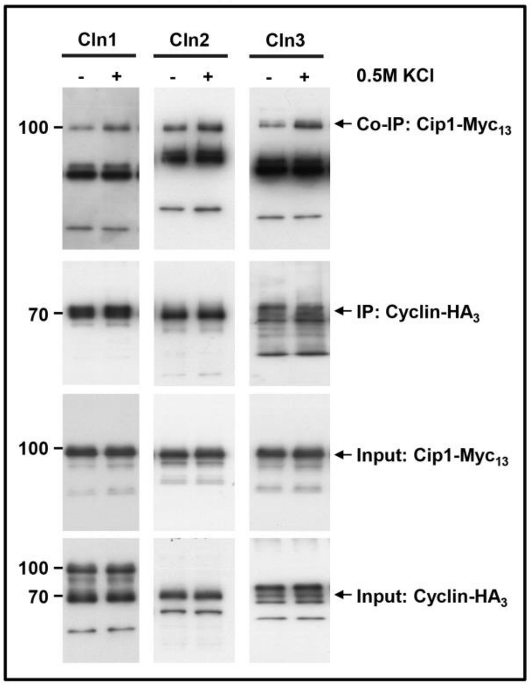

Figure 7b

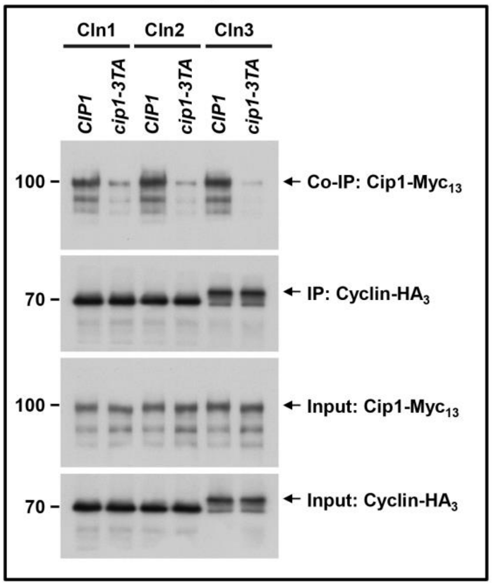

Figure 7d

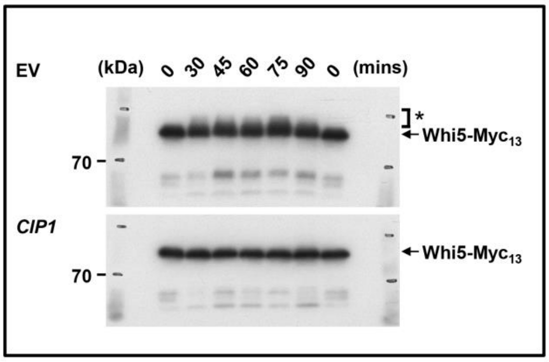

Figure 7e

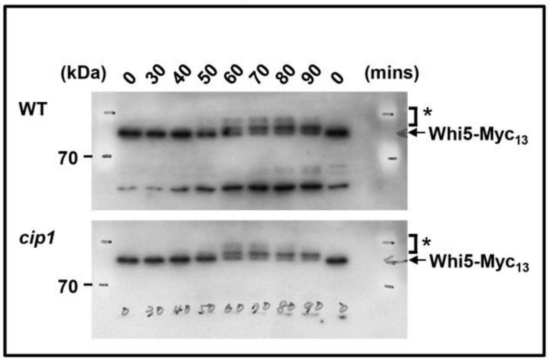

Figure 7g

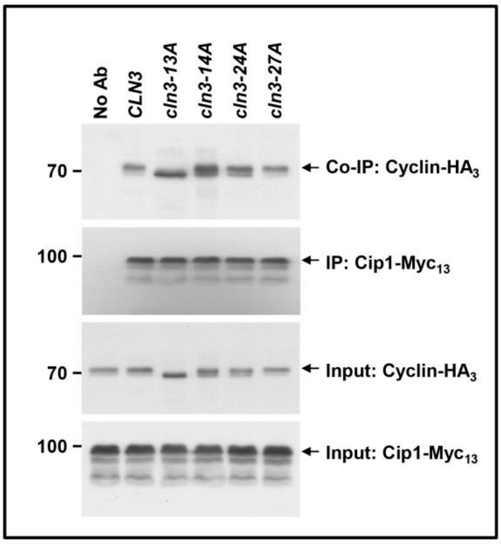

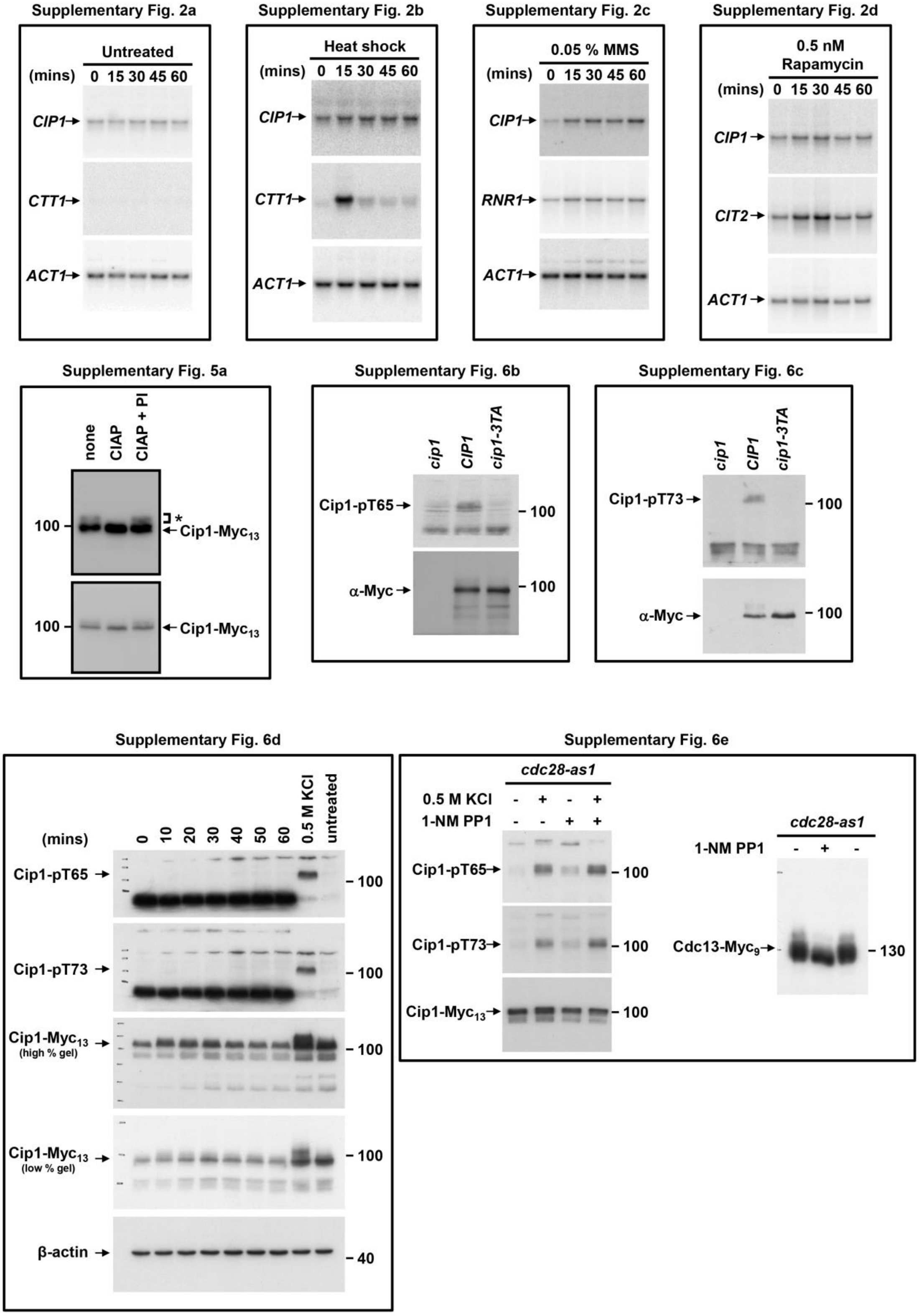

Supplementary Figure 11. Uncropped blots and gels displayed in the main

figures and supplementary information.

Supplementary Table 1. Strains Used in This Study.

Strain	Genotype	Source
BY4741	*MATα his3Δ1 leu2Δ0 met15Δ 0 ura3Δ 0*	Brachmann, C.B. *et al.*[2]
STY2998	BY4741 *msn2::KanMX4 msn4::KanMX4*	This study
STY3034	BY4741 *streI-CIP1*	This study
STY3035	BY4741 *streII-CIP1*	This study
STY3036	BY4741 *streI+II-CIP1*	This study
STY2996	BY4741 *ecbI+II-CIP1*	This study
	BY4741 *far1::KanMX4*	Deletion library
	BY4741 *sic1::KanMX4*	Deletion library
	BY4741 *cip1::KanMX4*	Deletion library
	BY4741 *ras1::KanMX4*	Deletion library
	BY4741 *ras2::KanMX4*	Deletion library
	BY4741 *gpa1::KanMX4*	Deletion library
	BY4741 *gpa2::KanMX4*	Deletion library
	BY4741 *tor1::KanMX4*	Deletion library
STY2984	BY4741 *far1::KanMX4 sic1::LEU2*	This study
STY2982	BY4741 *sic1::KanMX4 cip1:: HIS3MX6*	This study
STY3424	BY4741 *cip1-3TA*-Myc$_{13}$*::HIS3MX6*	This study

Strain	Genotype	Source
CHY125	*MATα ade2-1 ade3::hisG ura3-1 his3-12,15 trp1-1 leu2-3,112 can1-100*	Mullen, J.R. *et al.*[3]
STY2956	BY4741 *CIP1*-Myc$_{13}$*::HIS3MX6*	This study
STY3001	BY4741 *hog1::KanMX4 CIP1*-Myc$_{13}$*::HIS3MX6*	This study
STY3023	BY4741 *WHI5*-Myc$_{13}$*::KanMX4*	This study
YLY319	BY4741 *cip1::HIS3MX6 WHI5*-Myc$_{13}$*::KanMX4*	This study
BJ2168	*MATα leu2 trp1 ura3-52 pep4-3 prc1-407 prb1-1122 gal2*	Jones, E.W. *et al.*[4]
YLY348	BJ2168 *hog1::KanMX4*	This study
STY407	W303a *cdc28::cdc28 as-1* pRS304 *CDC13*-Myc$_{9}$	Tseng, S. F. *et al.*[1]
YLY398	W303a *cdc28::cdc28 as-1 bar1::hisG CIP1*-Myc$_{13}$::*His3MX6*	This study
YM4271	*MATa ura3-52 his3-Δ200 ade2-101 ade5 lys2-801 leu2-3,112 trp1-901 tyr1-501 gal4Δ gal80Δ ade5::hisG*	Wilson, T. E. *et al.*[5]

Supplementary Table 2. Constructs Used in This Study.

Name	Source
pEG(KT)-*CIP1*	This study
pEG(KT)-*cip1-3TA*	This study
pEG(KT)-*cip1-T65A*	This study
pEG(KT)-*cip1-T69A*	This study
pEG(KT)-*cip1-T73A*	This study
pEG(KT)-*cip1-T65AT69A*	This study
pEG(KT)-*cip1-T65AT73A*	This study
pEG(KT)-*cip1-T69AT73A*	This study
pEG(KT)-p21	This study
pGEX4T-1-*CIP1*	This study
pGEX4T-1-*msn2(401-704)*	Lee, P. *et al.*[6]
pYES2-*CLN1*-HA_3	This study
pYES2-*CLN2*-HA_3	This study
pYES2-*CLN3*-HA_3	This study
pYES2-*cln3-13A*-HA_3	This study
pYES2-*cln3-14A*-HA_3	This study
pYES2-*cln3-24A*-HA_3	This study

Name	Source
pYES2-*cln3-27A*-HA_3	This study
pYESL-*CIP1*-Myc_{13}	This study
pYEPFAT7-*CIP1*-GFP	This study
p*lacZi*-P^{CIP1} (-500~-1)	This study
p*lacZi*-P^{CIP1} *ecb I+II* (-500~-1)	This study
p*lacZi*-P^{CIP1} *stre I+II* (-500~-1)	This study

Supplementary Table 3. Primers Used in This Study

Primer	Sequence
Northern Blot	
ACT1-F	5’-GCCTTCTACGTTTCCATCCA-3’
ACT1-R	5’-GGCCAAATCGATTCTCAAAA-3’
RNR1-F	5’-GAGAGTCGCACTAGGCATCC-3’
RNR1-R	5’-TAGCAAACGGGCTTCCTCAG-3’
CTT1-F	5’-ATCCGGAATCAATCCATCAA-3’
CTT1-R	5’-GACGGGCAATTGCTGATAGT-3’
CIT1-F	5’-GGTTCCAACTCAAGCGCAAG-3’
CIT1-R	5’-ACCAAATGCCCTTGAAACGC-3’
qRT-PCR	
CIP1-RT-F	5’-GGCTCTTCTAGGCGATCTCA-3’
CIP1-RT-R	5’-CGGGAGTGGACAAGTTTCTG-3’
lacZ-RT-F	5’-CCGTTGATGTTGAAGTGGC-3’
lacZ-RT-R	5’-CTAATCCGAGCCAGTTTACCC-3’
ACT-RT-F	5’-ATGGATTCTGAGGTTGCTGCTTTGGTTA-3’
ACT-RT-R	5’-TGTTCTTCTGGGGCAACTCTCAATT-3’
Msn2 ChIP	

*CIP1*p-F	5'-ATGACGCTTGAACTCCGAAC-3'
*CIP1*p-R	5'-TTGTGCAATCTTTCCAGCAG-3'
TEL-F	5'-TAGTTGCACTAGGCGCAAAA-3'
TEL-R	5'-CAGCCCGCTTGTTAACTCTCC-3'
Mcm1 ChIP	
*CIP1*p-F	5’-GGAAGGGCGGCTTCTTACTA-3’
*CIP1*p-R	5’-TTCGGAGTTCAAGCGTCATT-3’
U2-F	5’- GGTTTGCTAAAGGGGAGGAG-3’
U2-R	5’-GCATTCTTCAAATCCCTCCA-3’
EMSA oligos	
STRE I-F	5’-GGAACTCGCGCAGGGGGAAGACATA-3’
STRE I-R	5’-TATGTCTTCCCCCTGCGCGAGTTCC-3’
STRE II-F	5’-GTATTTTCCTCCCCCTAGCTTCAAC-3’
STRE II-R	5’-GTTGAAGCTAGGGGGAGGAAAATAC-3’
stre I-F	5’-GGAACTCGCGCAGATGGAAGACATA-3’
stre I-R	5’-TATGTCTTCCATCTGCGCGAGTTCC-3’
stre II-F	5’-GTATTTTCCTCCATCTAGCTTCAAC-3’
stre II-R	5’-GTTGAAGCTAGATGGAGGAAAATAC-3’

Supplementary References

1. Tseng SF, Shen ZJ, Tsai HJ, Lin YH, Teng SC. Rapid Cdc13 turnover and telomere length homeostasis are controlled by Cdk1-mediated phosphorylation of Cdc13. *Nucleic Acids Res* **37**, 3602-3611 (2009).

2. Brachmann CB*, et al.* Designer deletion strains derived from *Saccharomyces cerevisiae* S288C: a useful set of strains and plasmids for PCR-mediated gene disruption and other applications. *Yeast* **14**, 115-132 (1998).

3. Mullen JR, Kaliraman V, Brill SJ. Bipartite structure of the SGS1 DNA helicase in *Saccharomyces cerevisiae*. *Genetics* **154**, 1101-1114 (2000).

4. Jones EW. Proteinase mutants of *Saccharomyces cerevisiae*. *Genetics* **85**, 23-33 (1977).

5. Wilson TE, Fahrner TJ, Johnston M, Milbrandt J. Identification of the DNA binding site for NGFI-B by genetic selection in yeast. *Science* **252**, 1296-1300 (1991).

6. Lee P, Cho BR, Joo HS, Hahn JS. Yeast Yak1 kinase, a bridge between PKA and stress-responsive transcription factors, Hsf1 and Msn2/Msn4. *Molecular microbiology* **70**, 882-895 (2008).
